# Complement receptor C5aR1 on osteoblasts regulates osteoclastogenesis in experimental postmenopausal osteoporosis

**DOI:** 10.3389/fendo.2022.1016057

**Published:** 2022-09-30

**Authors:** Jasmin Maria Bülow, Nikolai Renz, Melanie Haffner-Luntzer, Verena Fischer, Astrid Schoppa, Jan Tuckermann, Jörg Köhl, Markus Huber-Lang, Anita Ignatius

**Affiliations:** ^1^ Institute of Orthopedic Research and Biomechanics, Ulm University Medical Center, Ulm, Germany; ^2^ Institute of Comparative Molecular Endocrinology, University of Ulm, Ulm, Germany; ^3^ Institute for Systemic Inflammation Research, University of Lübeck, Lübeck, Germany; ^4^ Division of Immunobiology, Cincinnati Children’s Hospital Medical Center and University of Cincinnati College of Medicine, Cincinnati, OH, United States; ^5^ Institute of Clinical and Experimental Trauma-Immunology, Ulm University Medical Center, Ulm, Germany

**Keywords:** osteoblast, osteoclast, C5aR1, complement system, ovariectomy

## Abstract

In recent years, evidence has accumulated that the complement system, an integral part of innate immunity, may be involved in the regulation of bone homeostasis as well as inflammatory bone loss, for example, in rheumatoid arthritis and periodontitis. Complement may also contribute to osteoporosis development, but investigation of the mechanism is limited. Using mice with a conditional deletion of the complement anaphylatoxin receptor C5aR1, we here demonstrated that C5aR1 in osteoblasts (*C5aR1* ^Runx2-Cre^ mice) or osteoclasts (*C5aR1* ^LysM-Cre^ mice) did not affect physiological bone turnover or age-related bone loss in either sex, as confirmed by micro-computed tomography, histomorphometry, and biomechanical analyses of the bone and by the measurement of bone turnover markers in the blood serum. When female mice were subjected to ovariectomy (OVX), a common model for postmenopausal osteoporosis, significant bone loss was induced in *C5aR1* ^fl/fl^ and *C5aR1* ^LysM-Cre^ mice, as demonstrated by a significantly reduced bone volume fraction, trabecular number and thickness as well as an increased trabecular separation in the trabecular bone compartment. Confirming this, the osteoclast number and the receptor activator of nuclear factor k-B (RANK) ligand (RANKL) serum level were significantly elevated in these mouse lines. By contrast, *C5aR1* ^Runx2-Cre^ mice were protected from bone loss after OVX and the serum RANKL concentration was not increased after OVX. These data suggested that bone cell-specific C5aR1 may be redundant in bone homeostasis regulation under physiological conditions. However, C5aR1 on osteoblasts was crucial for the induction of bone resorption under osteoporotic conditions by stimulating RANKL release, whereas C5aR1 on osteoclasts did not regulate OVX-induced bone loss. Therefore, our results implicate C5aR1 on osteoblasts as a potential target for treating postmenopausal osteoporosis.

## Introduction

Bone is continuously renewed in a process termed bone remodeling, a dynamic balance of bone formation by osteoblasts and resorption by osteoclasts (1, 2). The main players in bone remodeling are osteocytes, which orchestrate osteoblast and osteoclast activity (3). Bone turnover is tightly regulated by a variety of factors, including hormones, growth factors, cytokines, and signaling transduction pathways (4, 5). A key mechanism regulating the interaction between osteoblasts and osteoclasts is the RANK/RANKL/OPG system (6). Receptor activator of nuclear factor k-B (RANK) ligand (RANKL), binds upon secretion by osteoblasts and osteocytes to the RANK receptor on osteoclast precursor cells, thereby inducing osteoclastogenesis. Osteoblasts and osteocytes also secrete osteoprotegerin (OPG), which acts as a decoy receptor for RANKL, thereby inhibiting osteoclast activity (7, 8). Many local and systemic factors which control bone homeostasis act *via* the modulation of the RANK/RANKL/OPG system (9). Imbalances in the regulation process can lead to bone disorders, including osteoporosis, one of the most prevalent disorders in the aged population. Osteoporosis is characterized by a progressive decline in bone mass, eventually resulting in fragility fractures, and is associated with a high morbidity, mortality, and socioeconomic burden (10, 11). There is increasing evidence that the immune system, which closely interacts with bone, plays a pivotal role in inducing bone loss under osteoporotic conditions, because osteoporosis is associated with low grade inflammation (12–15).

In recent years, evidence has accumulated that the complement system, a crucial component of the innate immune system, may be involved in physiological bone turnover as well as inflammatory bone disorders, including rheumatoid arthritis, periodontitis and bone fractures (16, 17). There are also indications that the complement system may contribute to osteoporosis development, because the blood levels of the complement factor C3, are increased in patients with postmenopausal osteoporosis (18, 19). Because C5a is generated downstream of C3 in the complement cascade, it can be suspected that the C5a/C5aR1 axis also plays a role in osteoporosis-associated inflammatory processes. Furthermore, complement-associated genes are up-regulated in osteoporotic bone (20). The complement system includes more than 40 proteases and is activated by one of the three main complement pathways, namely, the classical, lectin or alternative pathway. Activation of the complement system leads, amongst others, to the generation of the anaphylatoxin C5a. C5a is a strong chemoattractant and a potent pro-inflammatory mediator which, upon binding to its G protein-coupled membrane-bound receptor C5aR1, initiates inflammatory reactions by inducing degranulation, cytokine release and oxidative burst in immune cells (21, 22).

C5aR1 is also expressed by several non-immune cell populations, including osteoblasts and osteoclasts (23–25). Previous *in vitro* studies demonstrated that C5aR1 was greatly upregulated during osteogenic differentiation and that its activation by C5a induced osteoblast migration and the production of pro-inflammatory and osteoclastogenic factors, including RANKL, C-X-C motif chemokine ligand-10 (CXCL-10), interleukin (IL)-6 and IL-8 (23–26). This suggests that the C5a/C5R1 axis in osteoblasts may stimulate bone resorption *via* the RANK/RANKL/OPG system and by the release of other osteoclastogenic cytokines. Furthermore, C5R1 was shown to be expressed by osteoclasts and C5a could directly enhance osteoclast formation from hematopoietic precursor cells even in the absence of the key osteoclastogenic factor RANKL (26, 27). Notably, *in vitro* studies demonstrated an autocrine production of complement protein C5 by osteoblasts and its cleavage to biologically active C5a by osteoclasts (26). These data indicated that the C5a/C5aR1 axis may affect bone cell activity, their immune response and osteoblast-osteoclast crosstalk. Studies on bone homeostasis in murine models further corroborated these findings. Mice with a global deletion of C5aR1 (C5aR1^-/-^) displayed a high bone mass due to a reduced number of osteoclasts, implicating that the C5a/C5aR1 axis may also regulate osteoclast formation *in vivo* (28). However, it remains unclear whether the high bone mass phenotype of C5aR^-/-^ mice is due to a direct regulatory role of C5aR1 in bone cells or results from indirect effects caused, for example, by interactions of the immune and bone systems. Furthermore, it remains unclear whether the impact of C5a/C5aR1 on bone resorption is indirectly mediated by osteoblasts *via* the RANK/RANKL/OPG system or by a direct induction of osteoclast activity.

The aim of the present study was to elucidate the cell-specific role of the C5a/C5aR1 axis in the regulation of physiological bone turnover as well as in the pathophysiology of postmenopausal osteoporosis. Consequently, we generated mice with a cell-specific deletion of C5aR1 on osteoblasts and osteoclasts, respectively, and analyzed the bone phenotype at different ages and in either sex. To study the role of the C5a/C5aR1 axis in osteoporotic bone loss, we used a common model of postmenopausal osteoporosis, whereby female mice underwent ovariectomy (OVX) to induce estrogen decline, resulting in enhanced osteoclastogenesis and bone resorption.

## Material and methods

### Animal care and mouse models

All animal experiments were in compliance with international regulations for the care and use of laboratory animals (ARRIVE guidelines and EU Directive 2010/63/EU for animal experiments) and were approved by the responsible Local Ethical Committee (No. 1500, o.135-6 and o.135-10, Regierungspräsidium Tübingen, Germany). All animals were housed in groups of up to five mice per cage with a 12-hour light, 12-hour dark rhythm and received water *ad libitum* as well as a standard mouse feed (Ssniff R/M-H, V1535-300; Ssniff, Soest, Germany) until the day of OVX/sham-OVX. Thereafter, the mice received a phytoestrogen-free diet (Ssniff). Male and female mice with a cell-specific C5aR1 deletion in osteoblasts or osteoclasts were used for the study. Mice with an osteoblast- or osteoclast-specific C5aR1 deletion were generated by crossing floxed GFP-C5aR1-knock-in mice (B6.C5aR1tm1Jko) (29) with Runx2-Cre (Tg(Runx2-cre)1Jtuc) (*C5aR1* ^Runx2-Cre^) or LysM-Cre mice (Lyz2tm1(cre)Ifo) (*C5aR1* ^LysM-Cre^), respectively. Floxed mice with no Cre-transgene insertion were considered as controls (*C5aR1* ^fl/fl^). GFP-C5aR1-knock-in mice (B6.C5aR1tm1Jko) were kindly provided by Jörg Köhl (University of Lübeck, Lübeck, Germany). Runx2-Cre (Tg(Runx2-cre)1Jtuc) and LysM-Cre mice (Lyz2tm1(cre)Ifo) were kindly provided by Jan Tuckermann (Ulm University, Ulm, Germany). The genetic background of all mice was C57BL/6J. For genotyping, ear and tail biopsies were used and the DNA extraction was performed using the KAPA HotStart mouse genotyping kit (F Hoffmann-la Roche AG, Basel, Switzerland). Amplification of the different fragments was performed as previously described (29) using the following primers: Primer_01F: 5′-TAGAGTTGAGACTCAGAAAGACGG-3′, Primer_02R: 5′-GTACACGAAGGATGGAATGGTG-3′, Primer_03R: 5′-GGGTGGACAGGT-AGTGGTTATC-3′, and Primer_04R: 5′-CTCTTGT-TCTCTGT-TATCCAACCC-3′. The *LysM-Cre* transgene was detected using the primers: Primer_05F: 5′-ACCGTCAGTAGCTCACTAATCTT-3′, Primer_06R: 5′-ACCTGAAGATGTTCGCGATTA-TCT3′, Primer_07F: 5′-GAGACTCTGGCT-ACTCATCC-3′ and Primer_08R: 5′-CCTTCAGCA-AGAGCTGGGGAC-3′. The *Runx2-Cre* transgene was detected using the primers: Primer_09F: 5′-CCAGGAAGACTGCCAGAAGG-3′, Primer_10R: 5′-TGGCTTGCAGGTACAGGAG-3′ and Primer_11R: 5′-GGAGCTGCCGAGTCA-ATAAC-3′. Successful cell-specific C5aR1 deletion in osteoblasts or osteoclasts was confirmed by real-time PCR of primary cells isolated from long bones (osteoblasts) or bone marrow (osteoclasts) using the following primers: 5′-GGCCATCCTGCGGCTGATGG-3′ and 5′-GCCTTGCGA-CTCCAGGTCCG-3′. Additionally, the cell-specific knock out was determined on the protein level by immunofluorescence staining *in vitro* using the primary antibody rabbit anit-C5aR1 (1:50; #AP0658PU-N, Acris Antibodies, Herford, Germany) incubated for 72 h at 4°C For visualization, anti-rabbit immunglobulin G (IgG)-biotin (1:100; #B2770, Life Technologies, Carlsbad, CA, USA) and fluorescin-5-isothiocyanat-streptavidin (1:100; #405202, Biolegend, San Diego, CA, USA) were used and incubated for 1 h each at room temperature (RT). Nuclei were counterstained with Hoechst 33342 (1:1000) and osteoclast cytoskeleton was stained with rhodamin-phalloidin (1:140; Cytoskeleton, Denver, CO, USA). Images were obtained using a Leica microscope (DMI600B, Leica, Wetzlar, Germany) (**Supplemental Figures 1**, **2**).

### Bone phenotyping

To investigate the role of C5a/C5aR1 in osteoblasts or osteoclasts in physiological bone turnover, the skeleton of male and female mice aged 12 and 36 weeks was analyzed by biomechanical testing, micro-computed tomography (µCT) and histomorphometry, as described below.

### Ovariectomy

Female mice aged 12 weeks were randomly assigned to the different treatment groups either subjected to bilateral OVX as described previously (30) or sham operated (Sham). At 8 weeks post-surgery, all mice were euthanized using an isoflurane overdose and intracardiac blood withdrawal.

### Blood serum analyses

To determine the concentrations of bone turnover markers (procollagen type I N-terminal propeptide (PINP); OPG; C-terminal telopeptide (CTX); RANKL), blood was harvested at an age of 12 or 36 weeks and 8 weeks after OVX or sham operation in microvettes (Sarstedt AG & Co., Nümbrecht, Germany), centrifuged at 13,000 × g for 7 min for serum collection and stored at −80°C until further use. Bone turnover markers were determined using RatLaps™ CTX-I EIA, Rat/Mouse PINP EIA (both from Immunodiagnostic Systems GmbH, Frankfurt am Main, Germany), mouse RANKL ELISA Kit (TNFSF11), and mouse Osteoprotegerin ELISA Kit (TNFRSF11B) (both from Abcam, Cambridge, United Kingdom) according to manufacturers’ protocols. Using a customized mouse Multiplex Cytokine Kit (ProcartaPlex, eBiscience, Frankfurt, Germany), the CXCL-1, CXCL-10, IL-6, monocyte chemoattractant protein-1 (MCP-1), macrophage colony-stimulating factor (M-CSF), and vascular endothelial growth factor (VEGF) concentrations were determined in the OVX and Sham mice.

### Biomechanical testing

To analyze the mechanical bone properties, the flexural rigidity of the femurs was determined using a nondestructive three-point-bending test in 12- or 36-weeks old healthy mice and 8 weeks after OVX or Sham operation as previously described (31). Briefly, an axial load of 4 N was applied to the femoral midshaft, using a material-testing machine (Z10, Zwick Roell, Ulm, Germany). The flexural rigidity (EI) was calculated from the slope of the load-deflection curve (31).

### Micro-computed tomography

Femurs were fixed in 4% phosphate-buffered formaldehyde solution and scanned by µCT using a Skyscan 1172 (Bruker, Kontich, Belgium) at a resolution of 8 µm using a peak voltage of 50 kV and 200 µA. Analysis and calibration were performed according to the guidelines of the American Society for Bone and Mineral Research (ASBMR) (32). Bone mineral density was assessed using two phantoms with a defined density of hydroxyapatite (HA) (250 and 750 mg HA/cm^3^). The volume of interest (VOI) for cortical bone was set in the femur mid-diaphysis with a length of 168 µm at a threshold of 641.9 mg HA/cm^3^ (33). The VOI for the trabecular bone was defined in the distal part of the femur 200 µm proximal of the metaphyseal growth plate at the distal femur over a length of 280 µm with a threshold of 394.8 mg HA/cm^3^ (33).

### Histomorphometry and immunohistochemistry

Following the µCT scan, femurs were subjected to decalcified histology as previously described (34). Sections of 7 µm were stained for osteoblast and osteoclast quantification. Bone cells were evaluated 100 µm proximal to the metaphyseal growth plate using the Osteomeasure system (OsteoMetrics, Decatur, GA, USA). The number and surface of osteoclasts were quantitatively assessed by staining for tartrate-resistant alkaline phosphatase (TRAP). Osteoclasts were defined as TRAP-positive cells with two or more nuclei, directly located on the bone surface. Osteoblast number and surface were determined using toluidine blue staining. Osteoblasts were defined as cubic-shaped cells with visible cytoplasm, located directly on the bone surface. Images were obtained using a Leica microscope (DMI600B). All analyses were performed according to the ASBMR guidelines for histomorphometry (35).

For immunohistochemical analysis, 4 µm femur sections of the Sham/OVX mice were used. Staining for RANKL, CXCL-10, and IL-6 was performed using the following primary antibodies incubated overnight at 4°C: goat anti-mouse RANKL (1:100; # sc-7628, Santa Cruz, Dallas, TX, USA), goat anti-mouse CXCL-10 (1:50; #AF-466-NA, R&D Systems, Minneapolis, MN, USA), and rabbit anti-mouse IL-6 (1:250; #bs-0782R, Bioss, Woburn, MA, USA). As secondary antibodies, rabbit anti-goat IgG-biotin (1:200 and 1:300, respectively, for RANKL and CXCL-10 staining; #A10518, Invitrogen, Carlsbad, CA, USA) and goat anti-rabbit IgG-biotin (1:100; for IL-6; #B2770, Life Technologies) were used and incubated at RT for 30 minutes (IL-6) or 1 hour (RANKL and CXCL-10). For signal detection, horseradish peroxidase (HRP)-conjugated streptavidin (#PK-6100, VECTASTAIN Elite ABC-HRP Kit, Peroxidase, Vector Laboratories, Burlingame, CA, USA) was applied according to the manufacturer’s protocols. NovaRED (#SK-4800, Vector NovaRED Substrate Kit, Peroxidase, Vector Laboratories) was used as chromogen and the sections were counterstained with hematoxylin (1:2000, #2C-306, Waldeck, Münster, Germany). Images were obtained using a Leica microscope (DMI600B).

### Statistical analysis

The results were displayed as box and whisker plots (with median and interquartile range) from maximum to minimum, showing all data points. Depending on the experiment, we used 4- 9 mice per group and evaluation method, respectively. The sample sizes are indicated in the figure legends. Data in tables are expressed as mean ± standard deviation. Data were tested for normal distribution using Shapiro-Wilk normality test. Comparisons between the groups were performed by one-way analysis of variance (ANOVA) with Sidak multiple comparison test. Values of p<0.05 were considered as statistically significant. Statistical analysis was performed using Graph Pad Prism 8.0 software (GraphPad Software, La Jolla, CA, USA).

## Results

### Mice with osteoblast- or osteoclast-specific C5aR1 deletion did not display an altered bone phenotype

**Figure 1:**
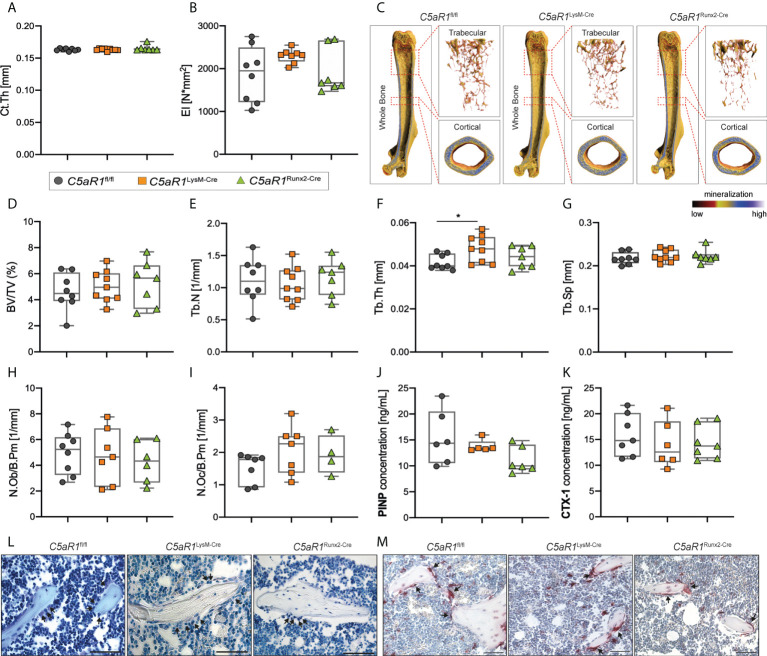
Bone phenotype of 12-week-old female mice with cell-specific C5aR1 deletion on osteoclasts (*C5aR1* ^LysM-Cre^) or osteoblasts (*C5aR1* ^Runx2-Cre^). **(A)** Cortical thickness (Ct.Th) and **(B)** flexural rigidity (EI) of the femurs. **(C)** Representative µCT images of the femurs with the trabecular and cortical compartments. **(D)** Relative bone volume (BV/TV) of the trabecular bone. **(E)** Trabecular number (Tb.N). **(F)** Trabecular thickness (Tb.Th). **(G)** Trabecular separation (Tb.Sp). **(H)** Number of osteoblasts (N.Ob/B.Pm) and **(I)** osteoclasts (N.Oc/B.Pm) per bone perimeter. **(J)** Concentration of procollagen type I N-terminal propeptide (PINP) and **(K)** C-terminal telopeptide (CTX-1) in the serum. **(L)** Representative images of toluidine blue-staining. Arrows indicate osteoblasts. **(M)** Representative images of tartrate-resistant acid phosphatase (TRAP)-staining. Arrows indicates osteoclasts. Scale bar 50 µm. *p<0.05, n=5–8 per group.

To investigate whether C5aR1 deletion on osteoblasts (*C5aR1* ^Runx2-Cre^) or osteoclasts (*C5aR1* ^LysM-Cre^) influences physiological bone turnover, we analyzed the bone phenotype of female and male mice aged 12 and 36 weeks using a three-point bending test, µCT and histomorphometry as well as serum analysis (**Figure 1**).

In 12-week-old female mice, the cortical thickness (Ct.Th) and flexural rigidity (EI) of the femurs were not significantly different in the mice with the bone cell-specific C5aR1 deletion compared to *C5aR1* ^fl/fl^ mice (**Figures 1A–C**). µCT analysis of the trabecular bone compartment also revealed no differences between female *C5aR1* ^LysM-Cre^ or *C5aR1* ^Runx2-Cre^ mice and *C5aR1* ^fl/fl^ mice in the bone volume fraction (BV/TV), trabecular number (Tb.N) or trabecular separation (Tb.Sp) (**Figures 1C–G**). Only the trabecular thickness (Tb.Th) of *C5aR1* ^LysM-Cre^ mice was slightly but significantly increased (**Figure 1F**), with no significant consequences on the bone mass or the other structural bone parameters. Consistent with these findings, no significant differences between *C5aR1* ^fl/fl^ and *C5aR1* ^Runx2-Cre^ or *C5aR1* ^LysM-Cre^ mice were observed in terms of osteoblast (N.Ob/B.Pm) or osteoclast numbers per bone perimeter (N.Oc/B.Pm) or their surface per bone surface (Ob.S/BS, Oc.S/BS) as well as the systemic concentrations of the bone turnover markers PINP or CTX (**Figures 1H–M**).

The 36-week-old female *C5aR1* ^LysM-Cre^ and *C5aR1* ^Runx2-Cre^ also displayed no major alterations in biomechanical, structural or cellular bone parameters. In both C5aR1 knockout strains, Tb.Th was slightly decreased compared to *C5aR1* ^fl/fl^ mice, whereas the BV/TV as well as all other measured parameters were not significantly changed (**Table 1**). As expected, a significant decrease in bone mass was observed in 36-week-old female *C5aR1* ^fl/fl^ mice compared to young mice, as confirmed by a significantly reduced BV/TV and Tb.N as well as increased Tb.Sp (**Table 1**). The age-dependent decrease of bone mass was also observed in 36-week-old *C5aR1* ^LysM-Cre^ and *C5aR1* ^Runx2-Cre^ mice compared to 12-week-old mice of the respective strains (**Table 1**).

**Table 1:** Bone phenotype of 36-week-old female mice.

	Parameters	*C5aR1* ^fl/fl^	*C5aR1* ^LysM-Cre^	*C5aR1* ^Runx2-Cre^
Cortical bone	EI (Nmm^2^)	2388 ± 158 ^$^	2711 ± 569 ^$^	2767 ± 555 ^$^
	Ct.Th (mm)	0.17 ± 0.00	0.16 ± 0.04	0.16 ± 0.01
Trabecular bone	BV/TV (%)	2.8 ± 1.4 ^$^	3.5 ± 1.7 ^$^	2.9 ± 1.8 ^$^
	Tb.Th (mm)	0.06 ± 0.01 ^$^	0.05 ± 0.01 *	0.04 ± 0.01 *
	Tb.N (1/mm)	0.50 ± 0.25 ^$^	0.74 ± 0.28 ^$^	0.63 ± 0.35 ^$^
	Tb.Sp (mm)	0.25 ± 0.01 ^$^	0.25 ± 0.01 ^$^	0.26 ± 0.03 ^$^
	N.Oc/B.Pm (1/mm)	1.0 ± 0.6	1.0 ± 0.3 ^$^	1.3 ± 0.4
	Oc.S/BS (1/mm)	2.5 ± 1.3 ^$^	3.4 ± 1.4 ^$^	3.5 ± 0.2 ^$^
	N.Ob/B.Pm (1/mm)	3.9 ± 1.5	3.1 ± 0.9	3.8 ± 0.4
	Ob.S/BS (1/mm)	2.1 ± 0.6	1.7 ± 0.7	2.9 ± 0.3

EI, flexural rigidity; Ct.Th, cortical thickness; BV/TV, bone volume per total volume; Tb.Th, trabecular thickness; Tb.N, trabecular number; Tb.Sp, trabecular separation; N.Oc/B.Pm, number of osteoclast per bone perimeter, Oc.S/BS, osteoclasts surface per bone surface; N.Ob/B.Pm, number of osteoblast per bone perimeter; Ob.S/BS, osteoblasts surface per bone surface. *p<0.05 compared to 36-week-old *C5aR1* ^fl/fl^ mice. ^$^ p<0.05 compared to 12-week-old mice of the respective strain (see **Figure 1**), n=4–9 per group. Bone phenotype was analyzed by three-point bending test, µCT and histological analyses.

Additionally, in male mice, C5aR1 deletion on osteoblasts or osteoclasts did not result in an altered bone phenotype (**Supplemental Tables 1**, **2**). Only the concentration of the bone turnover marker PINP was significantly decreased in 12-week-old male *C5aR1* ^Runx2-Cre^ mice compared to *C5aR1* ^fl/fl^ mice, whereas other parameters were not significantly affected (**Supplemental Table 1**). Similar to female mice, an age-dependent decrease of bone mass was observed in 36-week-old male mice, albeit the osteopenia phenotype was less pronounced compared to female mice (**Supplemental Table 2**). 36-week-old male *C5aR1* ^Runx2-Cre^ mice displayed a significantly reduced N.Oc/B.Pm and Oc.S/BS, but all other parameters were not significantly different compared to age-matched *C5aR1* ^fl/fl^ mice (**Supplemental Table 2**).

In summary, these results showed that mice with an osteoblast or osteoclast-specific C5aR1 deletion did not develop an altered bone phenotype at either age or in either sex, and that they underwent a physiological age-dependent decrease in bone mass similar to *C5aR1* ^fl/fl^ mice. This suggests that the C5a/C5aR1 axis in bone cells does not play an essential role in physiological bone turnover.

### Mice with osteoblast-specific C5aR1 deletion of were protected from OVX-induced bone loss

**Figure 2:**
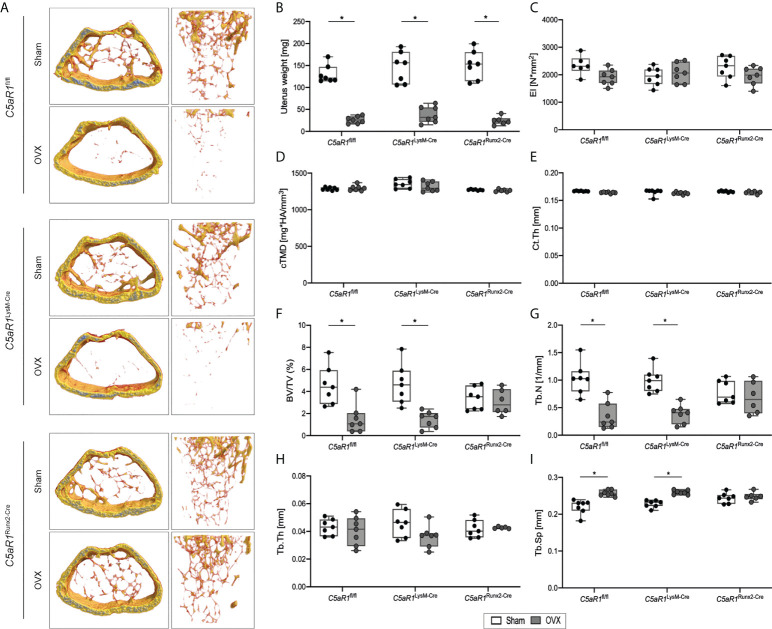
Bone phenotype of *C5aR1* ^LysM-Cre^ and *C5aR1* ^Runx2-Cre^ mice after Sham or OVX. **(A)** Representative µCT images of the trabecular bone of Sham operated and OVX mice. **(B)** Uterus weight of Sham operated and OVX mice 8 weeks post-surgery. **(C)** Flexural rigidity (EI) of the femurs. **(D)** Tissue mineral density of the cortex (cTMD) and **(E)** cortical thickness (Ct.Th) of Sham operated and OVX mice. **(F)** Relative bone volume (BV/TV) of the trabecular bone. **(G)** Trabecular number (Tb.N). **(H)** Trabecular thickness (Tb.Th). **(I)** Trabecular separation (Tb.Sp). *p<0.05, n=6–7 per group.

To investigate the role of the C5a/C5aR1 axis in bone cells in osteoporotic bone, female mice underwent OVX or sham operation. In all mouse strains, the uterus weight was significantly reduced by OVX, demonstrating estrogen deficiency (**Figure 2B**). The flexural rigidity (EI) of the femurs, cortical tissue mineral density (cTMD), and Ct.Th were not significantly affected by OVX in all mouse strains (**Figures 2C–E**). In ovariectomized *C5aR1* ^fl/fl^ and *C5aR1* ^LysM-Cre^ mice, trabecular bone mass was greatly reduced as compared to sham operated mice, confirmed by a significantly reduced BV/TV (−33.8% and −31.8%, respectively) and Tb.N (−31.1% and −38.4%, respectively) as well as significantly increased Tb.Sp (+19.3% and +14.5%, respectively), whereas Tb.Th was not changed (**Figures 2A, F−I**). By contrast, *C5aR1* ^Runx2-Cre^ mice were completely protected against OVX-induced trabecular bone loss (**Figures 2A, F−I**).

**Figure 3:**
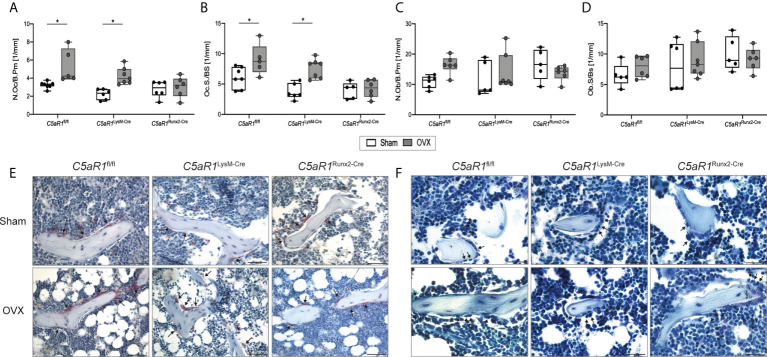
Cellular parameters in the trabecular bone of the femurs 8 weeks after Sham or OVX. **(A)** Number of osteoclasts per bone perimeter (N.Oc/B.Pm) and **(B)** osteoclast surface per bone surface (Oc.S/BS). **(C)** Number of osteoblasts per bone perimeter (N.Ob/B.Pm) and **(D)** osteoblast surface per bone surface (Ob.S/BS). **(E)** Representative images of tartrate-resistant acid phosphatase (TRAP)-staining. Arrows indicate osteoclasts. **(F)** Representative images of toluidine blue-staining. Arrows indicate osteoblasts. Scale bar 50 µm. *p<0.05, n=5–6 per group.

**Figure 4:**
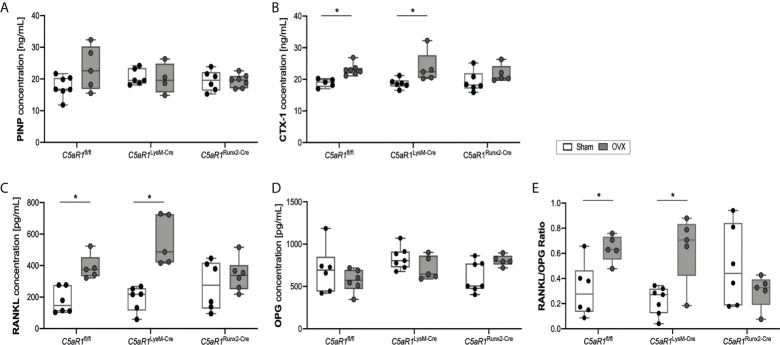
Serum concentration of bone markers 8 weeks after Sham or OVX. **(A)** Concentration of the bone markers procollagen type I N-terminal propeptide (PINP) and **(B)** C-terminal telopeptide (CTX-1) **(C)** Concentration of receptor activator of nuclear factor-kappa B ligand (RANKL) and **(D)** osteoprotegerin (OPG). **(E)** Ratio of RANKL/OPG. *p<0.05, n=5-7 per group.

Confirming this, the histological analysis revealed that N.Ob/B.Pm and Ob.S/BS as well as the serum concentration of the bone formation marker PINP remained unchanged after OVX in *C5aR1* ^fl/fl^ and *C5aR1* ^LysM-Cre^ mice, whereas the N.Oc/B.Pm and surface Oc.S/BS were significantly increased (**Figure 3**, **Figure 4A**), suggesting the decline in bone mass in these mouse strains was due to increased bone resorption. By contrast, in *C5aR1* ^Runx2-Cre^ mice, these osteoblast and osteoclast parameters were unaffected by OVX (**Figure 3**).

**Figure 5:**
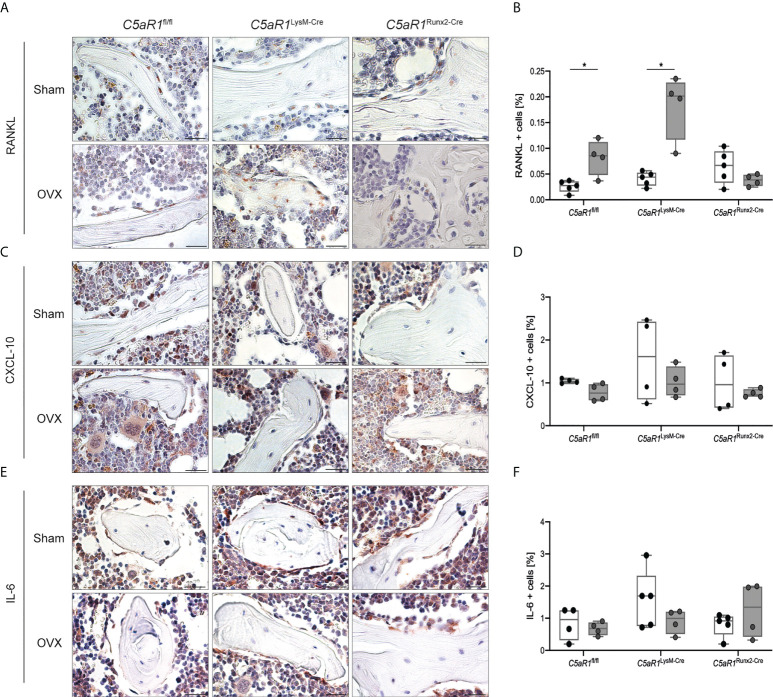
Quantification and representative immunohistological staining of cytokines in the trabecular bone of the femurs 8 weeks after Sham or OVX. **(A)** Representative staining of receptor activator of nuclear factor-kappa B ligand (RANKL) and **(B)** quantitative analysis of RANKL+ cells in the femur. **(C)** Representative staining of C-X-C motif chemokine 10 (CXCL-10) and **(D)** quantitative analysis of CXCL-10+ cells in the femur. **(E)** Representative staining of interleukin 6 (IL-6) and **(F)** quantitative analysis of IL-6+ cells in the femur. Scale bar 100 µm. *p<0.05, n=4–5 per group.

These results were further confirmed by the analysis of the bone turnover markers in the serum (**Figure 4**). In ovariectomized *C5aR1* ^fl/fl^ and *C5aR1* ^LysM-Cre^ mice, the serum concentrations of the bone resorption markers CTX-1 and RANKL were significantly elevated compared to Sham operated mice (**Figures 4B, C**). OPG concentrations were not affected (**Figure 4D**), resulting in a significantly increased RANKL/OPG ratio (**Figure 4E**). By contrast, *C5aR1* ^Runx2-Cre^ mice did not display OVX-induced alterations of bone resorption markers (**Figures 4B–E**). This was confirmed by increased numbers of RANKL+ osteoblasts in the femurs in ovariectomized *C5aR1* ^fl/fl^ and *C5aR1* ^LysM-Cre^ mice, whereas RANKL expression was not increased in *C5aR1* ^Runx2-Cre^ mice after OVX (**Figures 5A, B** *)*. The serum concentration of other cytokines, including CXCL-1, CXCL-10, IL-6, MCP-1, M-CSF, and VEGF, were not significantly affected after OVX in all mouse strains as well as the numbers of CXCL-10+ and IL-6+ cells in the bone (**Supplemental Table 3**; **Figures 5C–F**).

These data indicated that the C5a/C5aR1 axis in osteoblasts induces bone resorption *via* the RANK/RANKL/OPG system in OVX-induced bone loss, whereas C5aR1 on osteoclasts appears to be redundant in this process.

## Discussion

In this study, we demonstrated that mice with an osteoblast- or osteoclast-specific C5aR1 deletion did not develop an altered bone phenotype at any age or either sex. Furthermore, a similar age-dependent decrease in bone mass was observed in both knockout mouse strains as in control mice. These results suggested that the C5a/C5aR1 axis in osteoblasts and osteoclasts is redundant in physiological bone turnover. However, C5aR1 on osteoblasts may exert crucial functions in bone resorption when the system is challenged, because *C5aR1* ^Runx2-Cre^ mice were completely protected from bone loss induced by OVX, a murine model for postmenopausal osteoporosis. By contrast, *C5aR1* ^LysM-Cre^ mice developed OVX-induced osteoporosis similarly as *C5aR1* ^fl/fl^ mice, suggesting that C5aR1 on osteoclasts does not directly mediate increased bone resorption. Therefore, our results implicate C5aR1 on osteoblasts as a potential target for treating postmenopausal osteoporosis.

Several studies have shown that C5aR1 is expressed by osteoblasts and osteoclasts (16, 23, 26). It is greatly upregulated during osteoblastogenesis and mediates the production of osteoclastogenic cytokines, including the key mediator of osteoclastogenesis RANKL (26). C5aR1 is also expressed by osteoclasts and it was demonstrated that its stimulation could induce osteoclast formation even in the absence of RANKL *in vitro* (26, 27), suggesting that the C5a/C5aR1 axis in osteoblasts and osteoclasts may play a regulatory role in bone homeostasis. The *in vitro* results were further corroborated in mice with a global C5aR1 knockout, which displayed a high bone mass phenotype due to reduced osteoclast number and activity compared to wildtype controls, suggesting that C5aR1 may modulate physiological bone remodeling (28). By contrast, we showed in the present study that mice with a specific C5aR1 deletion in osteoblasts or osteoclasts do not develop a high bone mass phenotype or exhibit an altered age-dependent bone loss in either sex, suggesting that, under physiological conditions, the C5a/C5aR1 axis might not be essential for the regulation of bone turnover. It is, therefore, likely that the high bone mass phenotype observed in the global C5aR1 knockout mice used in the previous study (28) was not caused by bone-cell specific mechanisms, but rather by indirect effects, for example, by an altered immune cell function. Many immune cells which are known to closely interact with bone cells, including macrophages, neutrophils and T-cells (36–38), are crucially regulated by C5aR1 (39–41) and their function might had been affected by the general C5aR1 knockout.

However, the C5a/C5aR1 axis could play a crucial role in inflammatory bone loss, because C5aR1-deficient mice were protected against inflammatory arthritis (16, 42) and bone loss in periodontitis was found to be associated with increased C5aR1 activity (16, 43). Our group demonstrated that C5aR1 was upregulated by osteoblasts in response to bone injury and that mice with a global C5aR1 deletion exhibited disturbed bone fracture healing (28). We also showed that mice with an osteoblast-specific C5aR1 overexpression displayed a normal bone phenotype, whereas fracture healing in these mice was significantly disturbed due to high osteoclast numbers in the fracture callus, indicating that osteoblast-specific C5aR1 may regulate bone resorption (44). Moreover, preclinical and clinical data showed that the complement system may play a role in the development of postmenopausal osteoporosis (18–20, 45). C3-deficient mice displayed reduced bone loss after OVX compared to wildtype controls (45). Patients with postmenopausal osteoporosis exhibit increased blood levels of complement factors (18, 19) and complement-associated genes are up-regulated in osteoporotic bone (20). However, it remains unclear whether the C5a/C5aR1 axis specifically in bone cells plays a role in the development of postmenopausal osteoporosis, as no preclinical and clinical data are available so far. Therefore, we here investigated the function of the C5a/C5aR1 axis in OVX-induced bone loss. Furthermore, we aimed to clarify whether osteoclastogenesis is mediated directly by C5aR1 on osteoclasts, or indirectly *via* C5aR1-mediated secretion of osteoclastogenic factors by osteoblasts. As expected, OVX resulted in a significantly increased osteoclast activity and bone loss in *C5aR1* ^fl/fl^ mice, which was associated with an increased RANKL concentration and RANKL/OPG ratio (46–48). Similarly, mice with an osteoclast-specific deletion of C5aR1 developed osteoporosis after OVX due to increased osteoclast formation and activity. These data suggested that C5aR1 on osteoclasts does not play a crucial role during OVX-induced bone loss. Using bone marrow cell cultures, Tu et al. demonstrated that osteoclastogenesis essentially depended on the presence of C5aR, C3aR and C3 (27). Bone marrow cells isolated from C3^-/-^, C3aR^-/-^ and C5aR^-/-^ mice cultivated in the presence of 1,25(OH)_2_vitamine D_3_ showed a reduced osteoclast differentiation capacity compared to wildtype cells. Pharmaceutical inhibition of C3aR/C5aR activity also reduced osteoclast formation in bone marrow cell cultures (27). However, bone marrow cell cultures are heterogenous and contain stromal cells as well as cells of hematopoietic origin, preventing a clear discrimination of cell specific effects.

In contrast to *C5aR1* ^LysM-Cre^ mice, mice with an osteoblast specific deletion of C5aR1 were completely protected from bone loss after OVX. Osteoclast parameters were unchanged in *C5aR1* ^Runx2-Cre^ after OVX, suggesting that C5aR1 on osteoblasts regulates osteoclast number and activity. To understand the underlying molecular mechanism, we investigated various osteoclastogenic factors in the circulation as well as locally in the bone tissue. Whereas in ovariectomized *C5aR1* ^fl/fl^ and *C5aR1* ^LysM- Cre^ mice the RANKL/OPG ratio in the serum, the concentration of the bone resorption marker CTX-1 and the number of RANKL-positive osteoblasts in the bone tissue were significantly increased compared to sham operated mice, this was not the case in *C5aR1* ^Runx2-Cre^ mice, confirming our previous *in vitro* data, which showed that the C5a/C5aR1 axis in osteoblasts regulates RANKL expression and thus induces osteoclast formation (26, 44). We also measured additional pro-inflammatory and osteoclastogenic factors, which were shown to be regulated by C5a in osteoblasts, including IL-6 and CXCL-10. IL-6 is a strong inducer of osteoclastogenesis by stimulating the production of RANKL in osteoblasts (49, 50). IL-6 release was induced by the co-stimulation of osteoblastic cells with IL1-β/C5a (25, 26). Moreover, IL-6 expression was decreased in bone marrow cells from C5aR1-deficent mice and increased in mice with osteoblast-specific C5aR1 overexpression (27, 44). In the present study, IL-6 levels were not affected neither locally nor systemically by OVX in *C5aR1* ^fl/fl^ mice as well as in mice with bone cell-specific C5aR1 deletion. This is in contrast to previous studies demonstrating increased IL-6 concentrations in postmenopausal women (51, 52) and ovariectomized rodents (53, 54). In the latter studies, the animals were euthanized at earlier or later time points compared to the present study, which could be a reason for the different results. Therefore, the present study does not allow a final conclusion regarding the involvement of bone cell-specific C5aR1 in the regulation of IL-6 in OVX-induced bone loss. Another mediator shown to be regulated by C5a in osteoblasts is CXCL-10, which induces osteoclastogenesis by increasing RANKL expression in osteoblasts (55). We demonstrated previously that CXCL-10 expression was significantly enhanced after coactivation of C5aR1 and Toll-like receptor (TLR)-2, but not after isolated receptor stimulation (24). Here, we could not detect differences in CXCL-10 expression after OVX compared to sham animals of all mouse strains, indicating that an additional bacterial stimulus or TLR activation might be necessary to increase CXCL-10 expression (24, 56). In summary, our data indicated that C5aR1 on osteoblasts regulates osteoclastogenesis mainly *via* the RANK/RANK/OPG system during OVX-induced bone loss.

A limitation of this study is that we did not investigate the role of the second C5a receptor (C5aR2). C5aR2 is un-coupled from G-proteins and is generally described as a modulator of C5aR1 signaling, exerting either pro- or anti-inflammatory effects (57). Our group previously demonstrated that global C5aR2 deletion in mice resulted in an increased bone mass, albeit the phenotype was less pronounced compared to C5aR1^-/-^ mice (28). Interestingly, in contrast to C5aR1^-/-^ mice, the high bone mass in C5aR2^-/-^ mice was due to an increased osteoblast number and not a decreased osteoclast number (28). Therefore, we will address the cell specific role of C5aR2 in future studies. Another limitation of our study might be that we did not include older mice with a more pronounced age-related bone loss. This could be interesting, because we found a decrease of osteoclast numbers and surface in 36-week-old male *C5aR1* ^Runx2-Cre^ mice compared to age-matched controls, suggesting the possibility that the C5a/C5aR1 axis in osteoblasts also regulates osteoclast activity in old male mice. However, because all other measured bone parameters were not significantly different, this might be unlikely.

In conclusion, our results suggested that C5aR1 on osteoclasts or osteoblasts does not play a major regulatory role in physiological bone turnover. However, in ovariectomized mice, a common model for postmenopausal osteoporosis, C5aR1 on osteoblasts induces osteoclast activity by increasing the RANKL release. Therefore, our results implicate C5aR1 on osteoblasts as a potential target for the treatment of postmenopausal osteoporosis.

## Data availability statement

The original contributions presented in the study are included in the article/**Supplementary Material**. Further inquiries can be directed to the corresponding author.

## Ethics statement

The animal study was reviewed and approved by Regierungspräsidium Tübingen.

## Author contributions

JMB: conceptualization, investigation, visualization, data analysis, writing original draft. NR: investigation, data analysis. MeH-L: conceptualization, data analysis, writing original draft. VF: conceptualization, data analysis, AS: scientific discussion. JT: provided mouse models, scientific discussion. MaH-L: scientific discussion. JK: provided mouse models, scientific discussion. AI: conceptualization, writing original draft, supervision. All authors reviewed and approved the final version of the manuscript.

## Funding

The study was performed with support from the German Research Foundation (DFG; CRC 1149, ID 251293561, C01 INST 40/491-2).

## Acknowledgments

We thank Andrea Böhmler, Bettina Herde, Tina Hieber, and Gabriele Köhl for excellent technical assistance.

## Conflict of interest

The authors declare that the research was conducted in the absence of any commercial or financial relationships that could be construed as a potential conflict of interest.

## Publisher’s note

All claims expressed in this article are solely those of the authors and do not necessarily represent those of their affiliated organizations, or those of the publisher, the editors and the reviewers. Any product that may be evaluated in this article, or claim that may be made by its manufacturer, is not guaranteed or endorsed by the publisher.

## Supplementary material

Supplementary Figure 1 Cell-specific C5aR1 deletion on osteoclasts. **(A)** C5aR1 gene expression in primary osteoclasts at days 0 and 14 of differentiation. **(B)** Immunofluorescent staining of C5aR1 in primary osteoclasts of *C5aR1* ^fl/fl^ and C5aR1^LysM-Cre^ mice in green. Nuclei and F-actin are stained in blue and red, respectively. **(C)** PCR-based genotyping of C5aR1-fl/fl and **(D)** green fluorescent protein (GFP). **(E)** PCR-based control of successful C5aR1 deletion (C5aR1-Cre) and **(F)** cre-recombinase activity (LysM-Cre). The primer combination 05F:06R and 07F:08R amplifies a 609-bp DNA fragment in *C5aR1* ^fl/fl^ mice and a 370-bp DNA fragment in *C5aR1* ^LysM-Cre^ mice. Scale bar 50 µm. *p<0.05, n=6–9 per group.

Supplementary Figure 2 Cell-specific C5aR1 deletion on osteoblasts. **(A)** C5aR1 gene expression in primary osteoblasts at days 0 and 14 of differentiation. **(B)** Immunofluorescent staining of C5aR1 in primary osteoblasts of *C5aR1* ^fl/fl^ and *C5aR1* ^Runx2-Cre^ mice in green. Nuclei are stained in blue. **(C)** PCR-based genotyping of C5aR1-fl/fl and **(D)** GFP. **(E)** PCR-based control of successful C5aR1 deletion (C5aR1-Cre) and **(F)** cre-recombinase activity (*Runx2-Cre*). The primer combination 09F:10R/11R amplifies a 780-bp DNA fragment in *C5aR1* ^fl/fl^ mice and a 600-bp DNA fragment in *C5aR1* ^Runx2-Cre^ mice. Scale bar 50 µm. *p<0.05, n=5–8 per group.

Supplementary Table 1 Bone phenotype of 12-week-old male mice. Bone phenotype was analyzed by three-point bending test, µCT, and histological and serum analyses. EI: flexural rigidity, Ct.Th: cortical thickness, BV/TV: bone volume per total volume, Tb.Th: trabecular thickness, Tb.N: trabecular number, Tb.Sp: trabecular separation, N.Oc/B.Pm: number of osteoclast per bone perimeter, Oc.S/BS: osteoclasts surface per bone surface, N.Ob/B.Pm: number of osteoblast per bone perimeter, Ob.S/BS: osteoblasts surface per bone surface, CTX: C-terminal telopeptide, PINP: procollagen type I N-terminal propeptide. *p<0.05 compared to *C5aR1* ^fl/fl^ mice, n=5–9 per group.

Supplementary Table 2 Bone phenotype of 36-week-old male mice. Bone phenotype was analyzed by three-point bending test, µCT and histological analysis EI: flexural rigidity, Ct.Th: cortical thickness, BV/TV: bone volume per total volume, Tb.Th: trabecular thickness, Tb.N: trabecular number, Tb.Sp: trabecular separation, N.Oc/B.Pm number of osteoclasts per bone perimeter, Oc.S/BS: osteoclasts surface per bone surface, N.Ob/B.Pm: number of osteoblast per bone perimeter, Ob.S/BS: osteoblast surface per bone surface. *p<0.05 compared to *C5aR1* ^fl/fl^ mice. ^$^p<0.05 compared to 12-week-old mice of the respective strain (see **Supplemental Table 1**), n=5–8 per group.

Supplementary Table 3 Serum cytokine concentration after OVX in pg/mL. CXCL: C-X-C motif chemokine ligand, IL-6: interleukin-6, M-CSF: macrophage colony-stimulating factor, MCP-1: monocyte chemoattractant protein-1, VEGF: vascular endothelial growth factor. n=6–7 per group.
