# Supplementary figures and images for: Complement receptor C5aR1 on osteoblasts regulates osteoclastogenesis in experimental postmenopausal osteoporosis
Source: Front Endocrinol (Lausanne). 2022 Sep 30;13:1016057. doi: 10.3389/fendo.2022.1016057 (PMC9561253; doi:10.3389/fendo.2022.1016057)

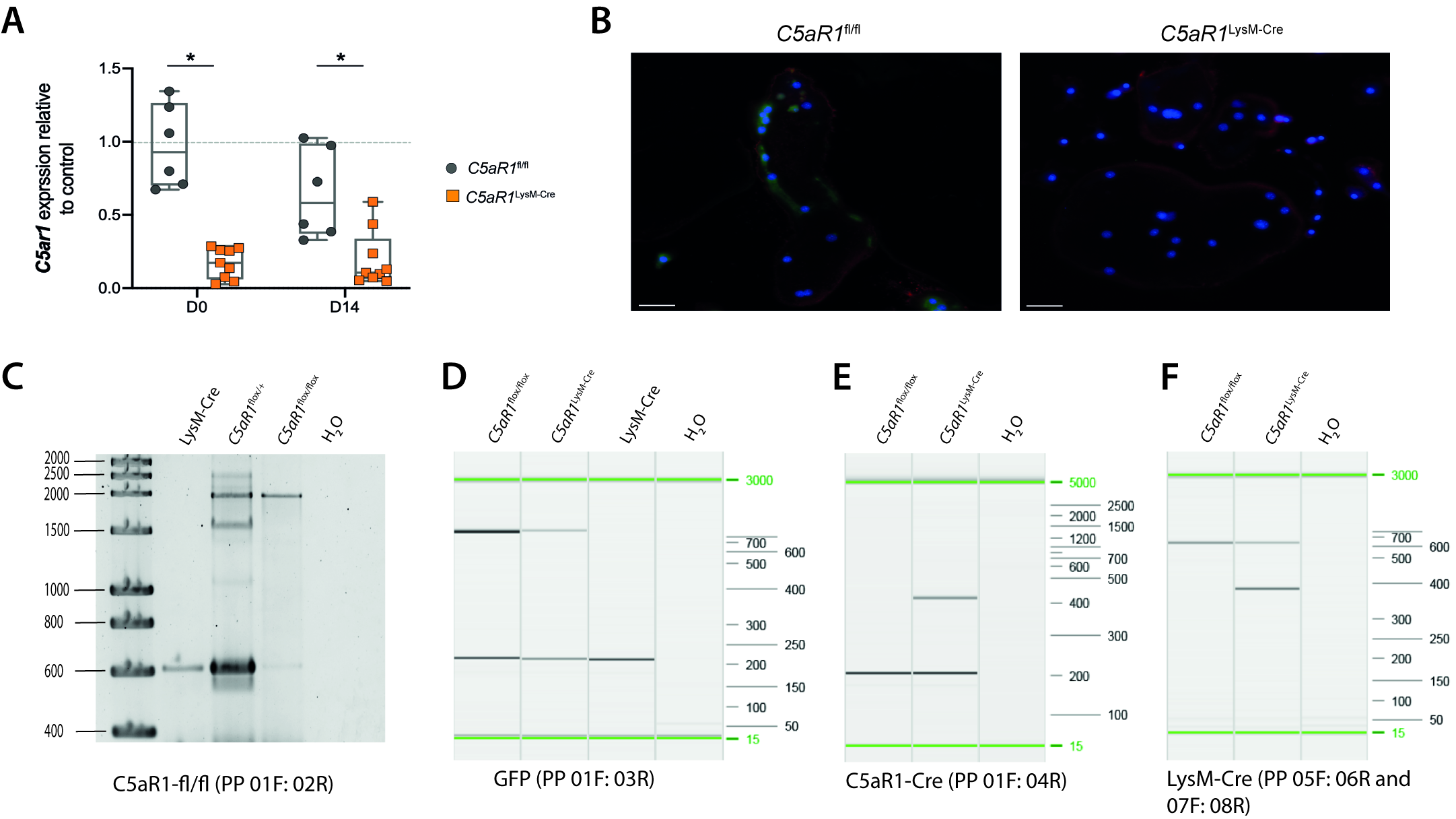

Supplement: Supplementary Figure 1 — Cell-specific C5aR1 deletion on osteoclasts. (A) C5aR1 gene expression in primary osteoclasts at days 0 and 14 of differentiation. (B) Immunofluorescent staining of C5aR1 in primary osteoclasts of C5aR1 fl/fl and C5aR1LysM-Cre mice in green. Nuclei and F-actin are stained in blue and red, respectively. (C) PCR-based genotyping of C5aR1-fl/fl and (D) green fluorescent protein (GFP). (E) PCR-based control of successful C5aR1 deletion (C5aR1-Cre) and (F) cre-recombinase activity (LysM-Cre). The primer combination 05F:06R and 07F:08R amplifies a 609-bp DNA fragment in C5aR1 fl/fl mice and a 370-bp DNA fragment in C5aR1 LysM-Cre mice. Scale bar 50 µm. *p<0.05, n=6–9 per group. [file Image_1.tif]

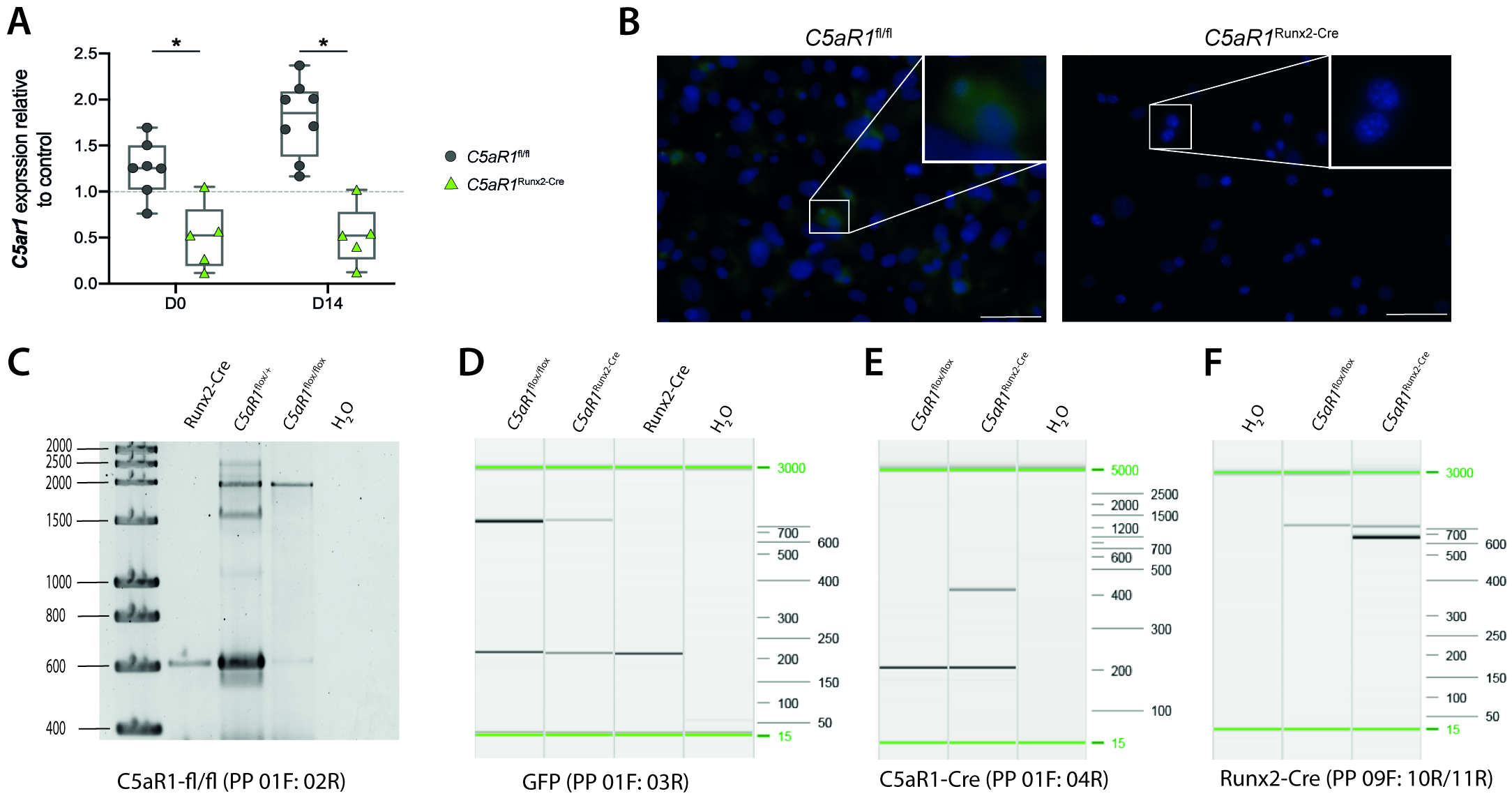

Supplement: Supplementary Figure 2 — Cell-specific C5aR1 deletion on osteoblasts. (A) C5aR1 gene expression in primary osteoblasts at days 0 and 14 of differentiation. (B) Immunofluorescent staining of C5aR1 in primary osteoblasts of C5aR1 fl/fl and C5aR1 Runx2-Cre mice in green. Nuclei are stained in blue. (C) PCR-based genotyping of C5aR1-fl/fl and (D) GFP. (E) PCR-based control of successful C5aR1 deletion (C5aR1-Cre) and (F) cre-recombinase activity (Runx2-Cre). The primer combination 09F:10R/11R amplifies a 780-bp DNA fragment in C5aR1 fl/fl mice and a 600-bp DNA fragment in C5aR1 Runx2-Cre mice. Scale bar 50 µm. *p<0.05, n=5–8 per group. [file Image_2.tif]
