# Supplementary material for: Complement receptor C5aR1 on osteoblasts regulates osteoclastogenesis in experimental postmenopausal osteoporosis
Source: Front Endocrinol (Lausanne). 2022 Sep 30;13:1016057. doi: 10.3389/fendo.2022.1016057 (PMC9561253; doi:10.3389/fendo.2022.1016057)
Supplement: Supplementary Table 1 — Bone phenotype of 12-week-old male mice. Bone phenotype was analyzed by three-point bending test, µCT, and histological and serum analyses. EI: flexural rigidity, Ct.Th: cortical thickness, BV/TV: bone volume per total volume, Tb.Th: trabecular thickness, Tb.N: trabecular number, Tb.Sp: trabecular separation, N.Oc/B.Pm: number of osteoclast per bone perimeter, Oc.S/BS: osteoclasts surface per bone surface, N.Ob/B.Pm: number of osteoblast per bone perimeter, Ob.S/BS: osteoblasts surface per bone surface, CTX: C-terminal telopeptide, PINP: procollagen type I N-terminal propeptide. *p<0.05 compared to C5aR1 fl/fl mice, n=5–9 per group. [file Table_1.pdf]

Supplemental Table 1: Bone phenotype of 12-week-old male mice. Bone phenotype was analyzed by three-point bending test, µCT, and histological and serum analyses. EI: flexural rigidity, Ct.Th: cortical thickness, BV/TV: bone volume per total volume, Tb.Th: trabecular thickness, Tb.N: trabecular number, Tb.Sp: trabecular separation, N.Oc/B.Pm: number of osteoclast per bone perimeter, Oc.S/BS: osteoclasts surface per bone surface, N.Ob/B.Pm: number of osteoblast per bone perimeter, Ob.S/BS: osteoblasts surface per bone surface, CTX: C-terminal telopeptide, PINP: procollagen type I N-terminal propeptide. *p<0.05 compared to *C5aR1*$^{fl/fl}$ mice, n=5–9 per group.

	**Parameters**	***C5aR1***$^{\mathbf{fl/fl}}$	***C5aR1***$^{\mathbf{LysM\text{-}Cre}}$	***C5aR1***$^{\mathbf{Runx2\text{-}Cre}}$
Cortical bone	EI (Nmm^2)	2507 ± 319	2459 ± 654	2554 ± 709
	Ct.Th (mm)	0.17 ± 0.00	0.18 ± 0.00	0.17 ± 0.01
Trabecular bone	BV/TV (%)	17.2 ± 3.1	16.5 ± 3.4	18.3 ± 1.3
	Tb.Th (mm)	0.06 ± 0.001	0.06 ± 0.00	0.06 ± 0.00
	Tb.N (1/mm)	3.1 ± 0.3	2.9 ± 0.5	3.3 ± 0.3
	Tb.Sp (mm)	0.17 ± 0.01	0.18 ± 0.01	0.17 ± 0.01
	N.Oc/B.Pm (1/mm)	1.04 ± 0.33	1.41 ± 0.53	1.34 ± 0.49
	Oc.S/BS (1/mm)	2.16 ± 0.76	3.10 ± 0.91 *	1.98 ± 0.62
	N.Ob/B.Pm (1/mm)	5.39 ± 1.64	4.99 ± 1.40	5.11 ± 1.06
	Ob.S/BS (1/mm)	3.28 ± 1.02	3.18 ± 1.01	2.78 ± 0.74
Serum	CTX (ng/mL)	15.6 ± 2.4	15.6 ± 2.7	17.1 ± 2.8
	PINP (ng/mL)	18.6 ± 5.4	14.8 ± 4.3	9.6 ± 3.5 *
