# Supplementary material for: Complement receptor C5aR1 on osteoblasts regulates osteoclastogenesis in experimental postmenopausal osteoporosis
Source: Front Endocrinol (Lausanne). 2022 Sep 30;13:1016057. doi: 10.3389/fendo.2022.1016057 (PMC9561253; doi:10.3389/fendo.2022.1016057)
Supplement: Supplementary Table 2 — Bone phenotype of 36-week-old male mice. Bone phenotype was analyzed by three-point bending test, µCT and histological analysis EI: flexural rigidity, Ct.Th: cortical thickness, BV/TV: bone volume per total volume, Tb.Th: trabecular thickness, Tb.N: trabecular number, Tb.Sp: trabecular separation, N.Oc/B.Pm number of osteoclasts per bone perimeter, Oc.S/BS: osteoclasts surface per bone surface, N.Ob/B.Pm: number of osteoblast per bone perimeter, Ob.S/BS: osteoblast surface per bone surface. *p<0.05 compared to C5aR1 fl/fl mice. $p<0.05 compared to 12-week-old mice of the respective strain (see Supplemental Table 1 ), n=5–8 per group. [file Table_2.pdf]

Supplemental Table 2: Bone phenotype of 36-week-old male mice. Bone phenotype was analyzed by three-point bending test, µCT and histological analysis EI: flexural rigidity, Ct.Th: cortical thickness, BV/TV: bone volume per total volume, Tb.Th: trabecular thickness, Tb.N: trabecular number, Tb.Sp: trabecular separation, N.Oc/B.Pm number of osteoclasts per bone perimeter, Oc.S/BS: osteoclasts surface per bone surface, N.Ob/B.Pm: number of osteoblast per bone perimeter, Ob.S/BS: osteoblast surface per bone surface. *$p<0.05$ compared to $C5aR1^{fl/fl}$ mice. $^{\$}p<0.05$ compared to 12-week-old mice of the respective strain (see Suppl. Tab. 1), n=5–8 per group.

	**Parameters**	$C5aR1^{fl/fl}$	$C5aR1^{LysM\text{-}Cre}$	$C5aR1^{Runx2\text{-}Cre}$
Cortical bone	EI (Nmm^2)	3469 ± 598 $^{\$}$	3634 ± 874 $^{\$}$	2706 ± 566
	Ct.Th (mm)	0.17 ± 0.00	0.16 ± 0.01	0.16 ± 0.00
Trabecular bone	BV/TV (%)	15.3 ± 4.5	15.3 ± 4.0	15.7 ± 4.7
	Tb.Th (mm)	0.07 ± 0.01 $^{\$}$	0.07 ± 0.01 $^{\$}$	0.06 ± 0.01
	Tb.N (1/mm)	2.20 ± 0.50 $^{\$}$	2.60 ± 0.50	2.60 ± 0.40 $^{\$}$
	Tb.Sp (mm)	0.20 ± 0.01 $^{\$}$	0.20 ± 0.01 $^{\$}$	0.20 ± 0.02 $^{\$}$
	N.Oc/B.Pm (1/mm)	1.5 ± 0.3 $^{\$}$	1.6 ± 0.5	0.7 ± 0.08 *
	Oc.S/BS (1/mm)	3.6 ± 1.3 $^{\$}$	2.5 ± 0.8	1.0 ± 0.2 *
	N.Ob/B.Pm (1/mm)	3.3 ± 0.9 $^{\$}$	3.7 ± 1.5	3.3 ± 1.3
	Ob.S/BS (1/mm)	1.6 ± 0.5 $^{\$}$	2.2 ± 0.8	2.0 ± 1.0
