# Supplementary material for: Complement receptor C5aR1 on osteoblasts regulates osteoclastogenesis in experimental postmenopausal osteoporosis
Source: Front Endocrinol (Lausanne). 2022 Sep 30;13:1016057. doi: 10.3389/fendo.2022.1016057 (PMC9561253; doi:10.3389/fendo.2022.1016057)
Supplement: Supplementary Table 3 — Serum cytokine concentration after OVX in pg/mL. CXCL: C-X-C motif chemokine ligand, IL-6: interleukin-6, M-CSF: macrophage colony-stimulating factor, MCP-1: monocyte chemoattractant protein-1, VEGF: vascular endothelial growth factor. n=6–7 per group. [file Table_3.pdf]

**Supplemental Table 3: Serum cytokine concentration after OVX in pg/mL.** CXCL: C-X-C motif chemokine ligand, IL-6: interleukin-6, M-CSF: macrophage colony-stimulating factor, MCP-1: monocyte chemoattractant protein-1, VEGF: vascular endothelial growth factor. n=6–7 per group.

| | ***C5aR1*$^{fl/fl}$** | | ***C5aR1*$^{LysM\text{-}Cre}$** | | ***C5aR1*$^{Runx2\text{-}Cre}$** | |
|---|---|---|---|---|---|---|
| | **Sham** | **OVX** | **Sham** | **OVX** | **Sham** | **OVX** |
| CXCL1 | 44.5 ± 29.5 | 41.9 ± 13.3 | 49.6 ± 15.5 | 59.2 ± 10.1 | 31.0 ± 6.3 | 43.2 ± 13.6 |
| CXCL10 | 58.9 ± 27.6 | 54.58 ± 9.8 | 81.9 ± 40.7 | 85.0 ± 19.7 | 55.7 ± 15.6 | 63.9 ± 17.3 |
| IL-6 | 17.8 ± 9.2 | 8.5 ± 6.5 | 9.0 ± 3.3 | 10.2 ± 9.0 | 11.7 ± 10.1 | 25.3 ± 32.0 |
| M-CSF | 1.7 ± 1.2 | 1.3 ± 0.3 | 2.0 ± 1.0 | 1.7 ± 0.6 | 4.6 ± 8.6 | 1.4 ± 0.4 |
| MCP1 | 34.7 ± 40.6 | 38.5 ± 43.0 | 18.5 ± 13.3 | 34.6 ± 35.4 | 15.5 ± 6.6 | 24.2 ± 25.5 |
| VEGF | 16.4 ± 6.7 | 15.4 ± 6.6 | 20.0 ± 3.8 | 18.7 ± 5.6 | 13.4 ± 4.7 | 14.8 ± 3.6 |
